# A pediatric wrist trauma X-ray dataset (GRAZPEDWRI-DX) for machine learning

**DOI:** 10.1038/s41597-022-01328-z

**Published:** 2022-05-20

**Authors:** Eszter Nagy, Michael Janisch, Franko Hržić, Erich Sorantin, Sebastian Tschauner

**Affiliations:** 1 Medical University of Graz, Department of Radiology, Division of Pediatric Radiology, Graz, Austria; 2 Medical University of Graz, Department of Radiology, Division of General Radiology, Graz, Austria; 3 University of Rijeka Faculty of Engineering, Department of Computer Engineering, University of Rijeka Center for Artificial Intelligence and Cybersecurity, Rijeka, Croatia

**Keywords:** Research data, Paediatric research, Diagnosis

## Abstract

Digital radiography is widely available and the standard modality in trauma imaging, often enabling to diagnose pediatric wrist fractures. However, image interpretation requires time-consuming specialized training. Due to astonishing progress in computer vision algorithms, automated fracture detection has become a topic of research interest. This paper presents the GRAZPEDWRI-DX dataset containing annotated pediatric trauma wrist radiographs of 6,091 patients, treated at the Department for Pediatric Surgery of the University Hospital Graz between 2008 and 2018. A total number of 10,643 studies (20,327 images) are made available, typically covering posteroanterior and lateral projections. The dataset is annotated with 74,459 image tags and features 67,771 labeled objects. We de-identified all radiographs and converted the DICOM pixel data to 16-Bit grayscale PNG images. The filenames and the accompanying text files provide basic patient information (age, sex). Several pediatric radiologists annotated dataset images by placing lines, bounding boxes, or polygons to mark pathologies like fractures or periosteal reactions. They also tagged general image characteristics. This dataset is publicly available to encourage computer vision research.

## Abstract (summary)

**Table:** 

Measurement(s)	wrist fracture • pronator quadratus sign • AO classifiction • soft tissue swelling • metal implant • osteopenia • plaster cast • bone Lesion • subperiosteal bone formation
Technology Type(s)	bone radiography

## Background & Summary

Wrist radiographs are routinely acquired to assess injuries around the wrist, distal forearm and the carpal bones, in the acute setting as well as in follow-up. This is also true in the pediatric population. Distal radius and ulna fractures account for the majority of pediatric wrist injuries with an incidence peak in adolescence^1–3^.

Pediatric surgeons in training or emergency physicians often interpret trauma radiographs, sometimes without being backed-up by experienced pediatric radiologists. Even in developed countries, shortages of radiologists were reported, posing a risk to patient care^4,5^. In some parts of the world, access to specialist reporting is considerable restricted, if not unavailable^6^.

Studies indicated various amounts of diagnostic error in reading emergency X-rays, up to a percentage of 26%^7–10^. Further modalities like ultrasound, computed tomography (CT), and magnetic resonance imaging (MRI) can be helpful in case of uncertainties after completed clinical examination and radiography. They increase sensitivity and specificity. However, some fractures are even occult in these modalities^11–13^.

Computer vision algorithms were found to be useful in many medical fields, mainly when large amounts of data can be analyzed. First positive experiences with the detection of pathologies in trauma X-rays were recently published^14–17^. There is also a limited number of studies dealing with automated detection of wrist fractures in adults^18–22^. Computer vision applications typically rely on large numbers of training samples^23,24^. Large publicly available datasets enable researchers around the globe to compare their algorithms in a standardized way, yet, there are still limited options accessible to date^25^.

We present a comprehensively annotated pediatric wrist trauma radiography dataset (GRAZPEDWRI-DX) for machine learning. The acronym is composed of the terms “***Graz***”, “***Ped***iatric”, “***Wri***st”, and “***D***igital ***X***-ray”. The image collection is de-identified, distributed in an accessible file format (Portable Network Graphics, PNG, 16-bit grayscale), and publicly available. It is accompanied by various annotations as well as image and object tags established by human experts.

## Methods

**Fig. 1:**
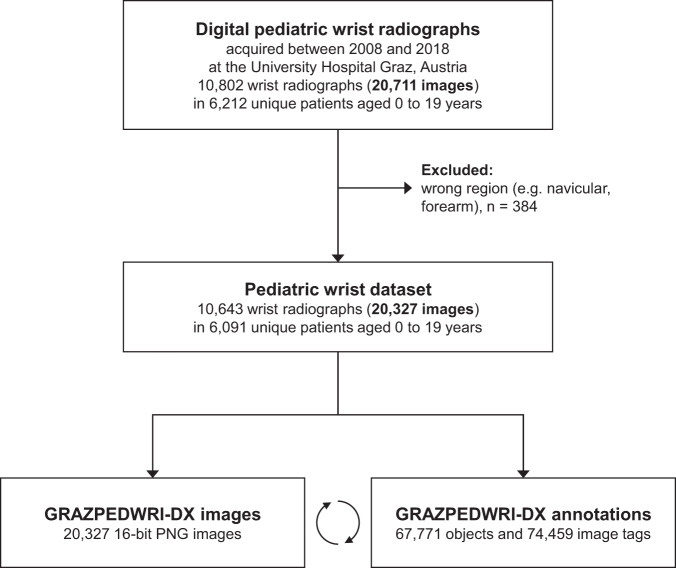
GRAZPEDWRI-DX dataset creation flowchart. Digital wrist radiographs were screened for eligibility in a pool of 20,711 images between 2008 and 2018. 384 images were excluded due to incorrect field of view that was not focused on the wrist. 20,327 images were included, tagged and annotated.

We constructed the GRAZPEDWRI-DX dataset from image data (pediatric wrist radiographs), natural language (report texts), and human expert annotations (bounding boxes, lines, polygons, and image tags). Annotations were established directly based onto the X-ray contents and with the aid of the corresponding free text reports, finally combined to the whole dataset. The whole process of dataset construction is presented as a flowchart in Fig. 1.

The project (No. EK 31-108 ex 18/19) was approved by the local ethics committee of the Medical University of Graz (IRB00002556). Requirement for oral or written patient consent was waived due to the retrospective project design. All study-related methods were conducted in accordance with the Declaration of Helsinki and all relevant guidelines and regulations.

### Pediatric wrist radiographs

10,643 wrist radiography studies of 6,091 unique pediatric patients (mean age 10.9 years, range 0.2 to 19 years; 2,688 females, 3,402 males, 1 unknown) were retrieved as Digital Imaging and Communications in Medicine (DICOM) images from the local Picture Archiving and Communication System (PACS). DICOM is the standard medical image format which enables permanent storage and exchange between different modalities and institutions^26,27^. Apart from the pixel information, DICOM images contain meta data (DICOM header) that rely on tight rules. Some of the meta data are mandatory, while others are optional. The DICOM standard is updated regularly^28^. Since there are still some barriers with the use of DICOM, we decided to convert the images to the more widespread Portable Network Graphics (PNG) format. PNG images offer a lossless compression of the pixel data and can be stored in more than 8 Bit grayscale format. Radiography DICOMs typically contain 12-bit, sometimes 16-bit of grayscale information. DICOM images served as input and were read with the “pydicom” package^29^. The PNG format does not allow to store 12 bits per channel, so we normalized the grayscale values of all DICOMs to 16-bit, stretching the grayscale histogram from initially 0 to 4,095 (12-bit) to 0 to 65,535 (16-bit) with the “cv2” module. Afterwards, PNGs were saved to the hard disk.

### Anonymization procedure

**Fig. 2:**
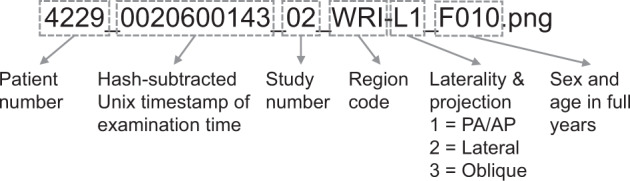
Meaning of the individual file name parts. File names for images and annotations start with a four-digit patient number, followed by a hashed examination time, the ascending study number, the region code (all “WRI”), the laterality and projection code, and finally sex and age in years.

De-identification and image conversion procedure was performed in batch by a custom-coded Python script. PNGs do not allow to store meta data as a standard feature. To retain sufficient information for processing and analysis of the PNG images, we created the file names based onto DICOM header information. The first part of the filename was constructed from concatenated strings of “Blake2b”^30^ cryptographic hashes of “Institution” and “Patient ID”, afterwards hashed with “SHA-3-256”^31^. The resulting hash is the same for each individual patient. The second part of the file name consisted of the acquisition time’s Unix timestamp, subtracted by a 9-integer long hash of the “Patient ID”, keeping the original study intervals while irreversibly masking the original examination times. The series number and image number (zero-padded to two digits) represented the next part of the filename, which was followed by a region token, the laterality and projection, and finally sex and age (rounded to one decimal place). An example file name would be: „e07bd2cecaefda782adff762c134e7f26f1b72dec670cb6c36d42ce40e3e30fb_0020600143_01-01_WRI-L1_F010-3.png“. For this specific publication, we replaced the ascending patient hashes by continuous numbers from zero onwards, forced to four digits by zero-padding, and provided only full years of age. Moreover, we subtracted the Unix timestamps by an arbitrary number a second time, and replaced series number and image number by ascending study IDs, e.g. „4229_0020600143_02_WRI-L1_F010.png“, explained in Fig. 2.

### DICOM layers and overlays

Any additional layers or overlays in the original DICOM were irreversibly discarded during the image conversion and de-identification process.

### Burned in annotations

After DICOM conversion including removal of the header information, PNG images can still contain identifying text in their pixel matrix, usually referred to as “Burned In Annotation”. These text passages, written into the image pixels can contain sensitive information about the patient, the radiological technologists or even the referring clinician. We found our DICOM headers routinely inaccurate regarding the presence of the “Burned In Annotation” attribute. Automated text-recognition methods based on the Tesseract optical character recognition software were not reliable enough to enable unvalidated masking of potentially sensitive information. We therefore screened all images manually and masked related images with black boxes in IrfanView (n = 97). In contrast to other publicly available datasets, we also masked radiographer abbreviations.

### Image labeling

Board-certified pediatric radiologists (S.T., E.N., and E.S.) with experiences between 6 and 29 years in musculoskeletal radiology validated all images annotations performed on the Supervisely (Deep Systems LLC, Moscow, Russia) artificial intelligence online platform. A dedicated server hosted the web-based image database, allowing for collaborate labeling. Clients accessed the server with a web browser over the Internet. Apart from the mentioned validating radiologists, local radiologists, visiting colleagues, and medical students helped to progress the dataset with different shares of labeling times. All annotations were executed between March 2018 and February 2022.

**Fig. 3:**
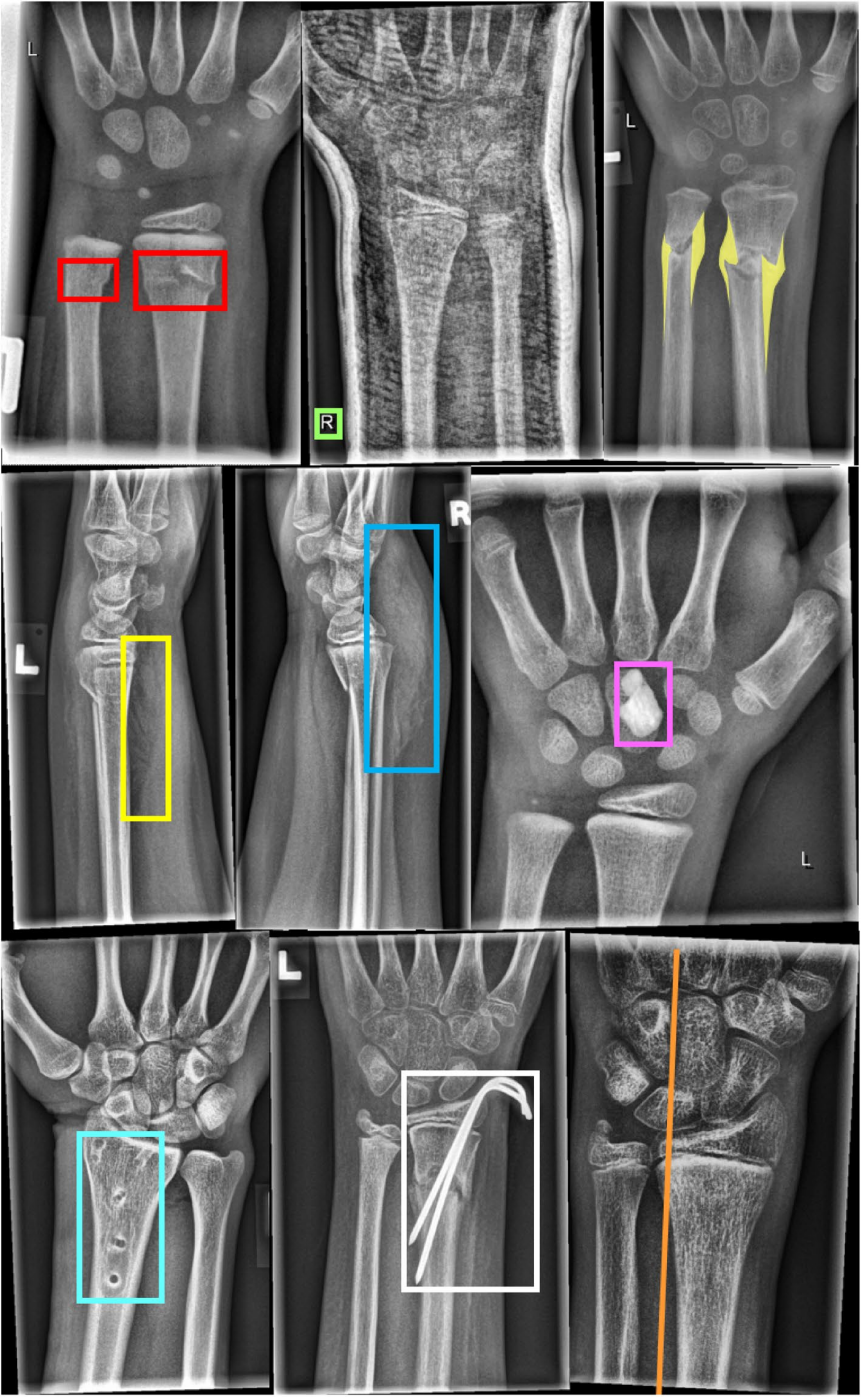
Examples of different objects labelled by the human experts. In the first row from left to right: Fracture (bounding box), text (bounding box), periosteal reaction (polygon). In the second row from left to right: Pronator quadratus sign (bounding box), soft tissue swelling (bounding box), foreign body (bounding box). In the last row from left to right: Bone anomaly (bounding box), metal (bounding box), and axis (line). The middle radiograph in the first row shows a cast. The right images in the first and third row were tagged with osteopenia.

The annotators manually placed objects with dedicated tools. Amongst others, we annotated fractures, metal implants, periosteal reactions, or bone lesions with bounding boxes, polygons, or lines. Image tags were manually set to represent features of each image, when appropriate. Collected objects (Fig. 3.) and image tags are listed in Tables 1 and 2.

**Table 1:** Numbers and percentages of annotated objects.

Object	Type	Object count	One or more objects present in the image
“axis”	Line	n = 20,327 (29.99%)	n = 20,327 (100.00%)
“boneanomaly”	Box	n = 276 (0.41%)	n = 192 (0.94%)
“bonelesion”	Polygon	n = 45 (0.07%)	n = 42 (0.21%)
“foreignbody”	Box	n = 8 (0.01%)	n = 8 (0.04%)
“fracture”	Box	n = 18,090 (26.69%)	n = 13,550 (66.66%)
“metal”	Box	n = 819 (1.21%)	n = 708 (3.48%)
“periostealreaction”	Polygon	n = 3,453 (5.10%)	n = 2,235 (11.00%)
“pronatorsign”	Box	n = 567 (0.84%)	n = 566 (2.78%)
“softtissue”	Box	n = 464 (0.68%)	n = 439 (2.16%)
“text”	Box	n = 23,722 (35.00%)	n = 20,274 (99.74%)
**Total**		**n = 67,771 (100.00%)**	**n = 20,327 (100.00%)**

Data are given in total and based on presence in a single image. Object descriptions are presented in the text. Each image can be assigned with multiple objects.

**Table 2:** List of tags associated with the images.

Image tag	Description	Count
“ao_classification”	AO classification code	n = 14,158 (69.65%)
“cast”	Cast/plaster present	n = 5,776 (28.42%)
“diagnosis_uncertain”	Fracture labels / tags uncertain	n = 537 (2.64%)
“initial_exam”	Initial trauma presentation	n = 10,861 (53.43%)
“metal”	Presence of metal implants	n = 708 (3.48%)
“osteopenia”	Signs of osteopenia	n = 2,473 (12.17%)
“projection_ap”	Anteroposterior projection view	n = 10,086 (49.62%)
“projection_lat”	Lateral projection view	n = 10,148 (49.92%)
“projection_oblique”	Oblique projection view	n = 93 (0.46%)
“side_left”	Left side	n = 11,135 (54.78%)
“side_right”	Right side	n = 9,192 (45.22%)
**Total**		n = 20,327 (100.00%)

Tag shortcuts, descriptions, and number of related images are presented. There can be one tag per image.

### Pediatric radiology reports

All available radiology reports were manually read by the authors and classified either into fracture or no fracture. They served as basis for labeling fractures in the annotation period. The German natural language reports are not delivered together with the dataset as 1) we were not able assure full anonymity, 2) they might not be correctly linked to the corresponding image occasionally due to pooling of images of different date and/or body regions into single free text reports. However, we are willing to provide the reports for research purposes on request.

## Data Records

Data are provided for download on Figshare (10.6084/m9.figshare.14825193)^32^. We grant free access to the dataset, without the need for user registration. The dataset is distributed in ZIP archives with a total size of 15.2 Gigabytes (GB), containing its original folder structure displayed below.

### Folder structure

We provide ready-to-use data in proprietary Supervisely project (“supervisely” folder) format, in PASCAL Visual Object Classes (VOC)^33^ format (“pascalvoc” folder), in YOLOv5^34^ format (“yolov5” folder), as well as image tags in comma separated values (CSV) format. The whole project structure is shared by a ZIP file.

The root folder contains a CSV file (“dataset.csv”) with all filenames, basic patient information and image tags. The “notebooks” folder holds three pieces of code, one for previewing annotations, and one for splitting files based on two columns of a CSV file, and one image post-processing. They are released as Jupyter notebooks. The “images” folder contains all wrist radiographs in 16-bit PNG format. The “supervisely” folder has a single “meta.json” file and is structured into a “wrist” subfolder that consists of an “ann” subfolder with json annotations, and an empty “img” subfolder. This “img” subfolder needs to be populated from the “images” folder, if needed. The “pascalvoc” folder contains Extensible Markup Language (XML) data files for object detection. This folder also does not include image files, which need to be copied from the “images” folder, if necessary. Every annotation file is associated with a corresponding image file, featuring the same file basename and only variable file extension. The “yolov5” folder contains the labels as text files (TXT) in the dedicated YOLOv5 format. It comes with a “meta.yaml” file for basic settings and object mappings.

**Fig. 4:**
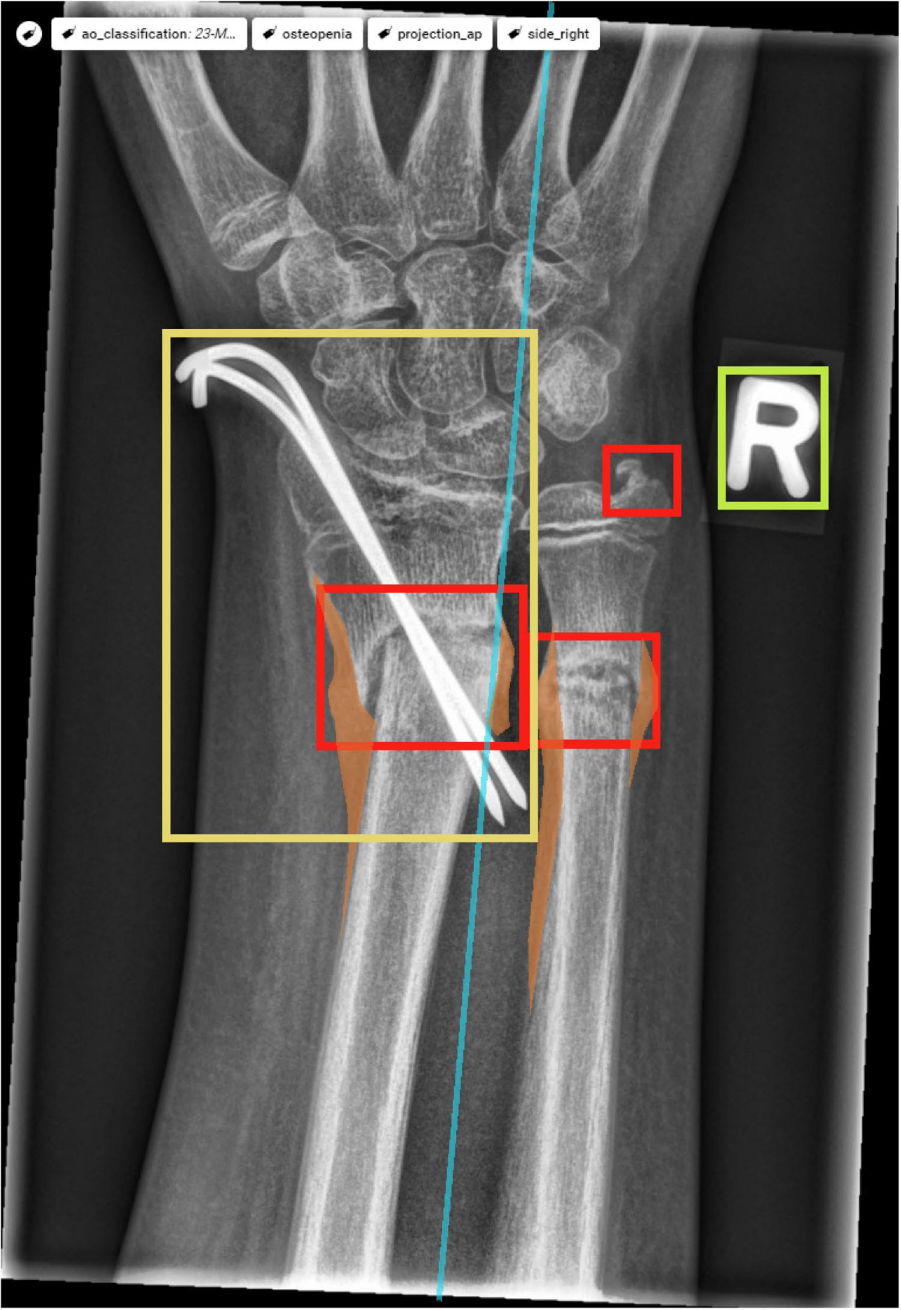
Fully tagged and labelled sample image viewed in the Supervisely online platform.

An annotated example study is provided in Fig. 4.

A total number of 67,771 objects were annotated in the dataset, splitting into the different categories as specifically detailed in Table 1. The “axis object consist of a two-point line along the main axis of the forearm bones. It is thought to be of help in automatically aligning the images. Bone anomalies (“boneanomaly”) are a heterogenous group of objects, representing bone pathologies from drill holes to Madelung’s deformities. Bone lesions (“bonelesion”) are bone tumors like osteomas. “foreignbody” refers to objects that label foreign bodies visible onto the image. The “fracture” object is used to annotate fractures. The “metal” object is used for internal or external metal implants. The annotators labeled periosteal reactions and callus under the label “periostealreaction” with polygons. The “pronatorsign” annotation resembles the specific radiological sign in terms of a swelling in the Musculus pronator quadratus region^35,36^. “softtissue” refers to an unspecific soft tissue swelling, usually due to trauma. “text” marks any text passage or single letters visible, most commonly laterality indicators “L” and “R”.

The annotators set 74,459 image tags in the 20,327 images. They included AO/OTA (“Arbeitsgemeinschaft für Osteosynthesefragen” /”Orthopaedic Trauma Association”) classifications (2018 version)^37^ in images with acute or subacute fractures, but not in healed or remodeled fractures. Images might contain AO classifications without a labeled fracture object (bounding box) in cases, where a fracture is known to be present but not clearly visualized in the respective projection. Table 2 lists and describes the different image classifications.

Top 5 most common AO classification ratings in the dataset are “23r-M/2.1” (n = 3,221), “23r-M/3.1; 23u-M/2.1” (n = 1,683), “23r-M/3.1; 23u-E/7” (n = 1,529), “23r-M/3.1; 23u-M/2.1” (n = 1,501), and “23-M/3.1” (n = 1,436), followed by a number of 102 further AO classifications or combinations that can be retrieved from the accompanying CSV file. Multiple fracture types within the same image are separated by semicolons.

## Technical Validation

**Fig. 5:**
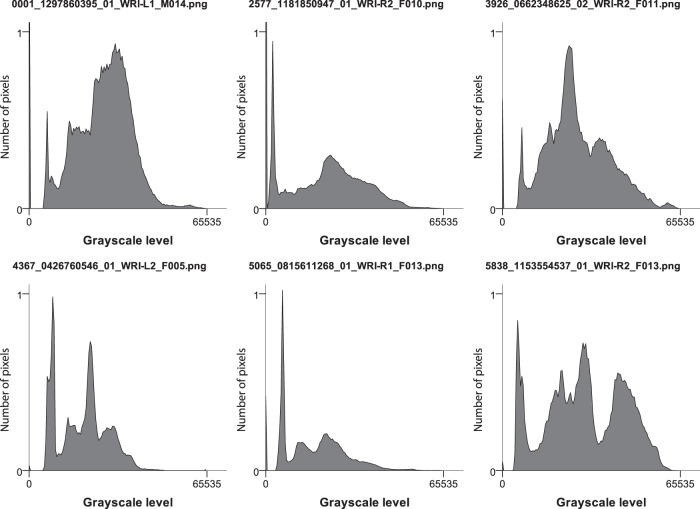
Histograms of 6 sample images showing their grayscale spectra. Note that the full grayscale range was preserved when converting and normalizing the images. The relative number of pixels per grayscale level are normalized from 0 to 1 for display purposes.

Image data is of high quality, delivered in full pixel resolution and complete grayscale spectrum. We did not apply any post-processing apart from histogram normalization (stretching) the usually 12-bit input spectrum to 16-bit due to meet PNG image format requirements. Note that there is no loss of information due this reversible procedure (Fig. 5.). Postprocessing is advised for typical computer vision tasks. We attached a script to optimize image contrast and convert the gray levels to 8 bits (*“image_conversion.ipynb”*).

All studies and their annotations were reviewed by experienced pediatric radiologists at least twice. Still, users need to consider inevitable inaccuracies, discrepancies, and errors in labeling due to the restricted diagnostic sensitivity of X-ray studies^38,39^. Images in question are tagged “diagnosis_uncertain” (n = 537, 2.64%). However, it must be kept in mind that annotation errors might not be confined to these images, but could be present within the whole dataset. Particularly AO classification codes are not always unambiguous. Images that are considered normal might contain occult fractures, while we believe that studies rated to be pathologic, are accurately tagged in the majority of cases.

We assessed “fracture” labeling agreement between the validating pediatric radiologists with intersection over union (IoU) measurements in a subsample of 100 randomly chosen overlapping images, resulting in a mean IoU 0.70 (1^st^ percentile 0.22, 25^th^ percentile 0.63, 50^th^ percentile = median 0.73, 75^th^ percentile 0.80, 99^th^ percentile 0.94 for the blinded raters. An IoU of 1 resembles perfectly overlapping bounding boxes, while 0 would be no overlap at all. Thus, inter-rater bounding box agreement was moderate. However, the whole dataset was labeled in an iterative continuous fashion, where labels were little-by-little adjusted multiple times by different annotators. So labeling quality is considered more accurate than the IoU level alone might indicate.

### Object detection performance

We trained a state-of-the-art neural network for object detection, namely YOLOv5^34^ by randomly dividing the total dataset into a training set of 15,327 (of 20,327), and a validation set of 4,000 images. A Windows PC equipped with an Nvidia GeForce RTX 2060 SUPER (video memory size of 8,192 MB) ran 50 epochs of a COCO^40^ pre-trained “YOLOv5m” model with an input size of 640 pixels and a batch size of 16 samples. Standard hyperparameters were used. Python version was 3.9.5. Table 3 lists the results (precision, recall, mean average precision = mAP) achieved on the previously unseen test subset of 1,000 random samples. Fractures were detected with a precision of 0.917, a recall of 0.887, and a mAP of 0.933 at an IoU threshold of 0.5.

**Table 3:** Results of baseline object testing with a pre-trained YOLOv5 model.

Class	Images	Labels	Precision	Recall	mAP@0.5	mAP @0.5:0.95
“boneanomaly”	1,000	15	0.633	0.267	0.386	0.158
“bonelesion”	1,000	3	0.000	0.000	0.012	0.009
“foreignbody”	1,000	2	1.000	0.000	0.496	0.446
“fracture”	1,000	911	0.917	0.887	0.933	0.544
“metal”	1,000	33	0.918	1.000	0.986	0.777
“periostealreaction”	1,000	175	0.713	0.64	0.675	0.317
“pronatorsign”	1,000	32	0.835	0.656	0.82	0.378
“softtissue”	1,000	27	0.397	0.22	0.302	0.139
“text”	1,000	1,170	0.975	0.987	0.992	0.731
Total	1,000	2,368	0.71	0.517	0.622	0.389

## Usage Notes

The dataset is made freely available for any purpose. The data provided within this work are free to copy, share or redistribute in any medium or format. The data might be adapted, remixed, transformed, and built upon. The dataset is licensed under a Creative Commons “Attribution 4.0 International” license (CC BY 4.0) (https://creativecommons.org/licenses/by/4.0/). Users are prohibited to attempt re-identification of personal patient information.

Correct use of the dataset requires medical and radiological background knowledge, especially in interpreting obtained results and in drawing dataset-based conclusions. Potential labeling errors must be taken into consideration, as pointed out in the “Technical Validation” section. Our key aim in releasing this dataset is to encourage research in automated pediatric fracture detection. We believe that releasing the GRAZPEDWRI-DX dataset could improve research in this field.

Computer vision algorithms commonly rely on input images with 8 bits per channel for training and inference, particularly when using transfer learning bases on large image archives. The dataset contains a Python script that enables image conversion from the 16-bit source images to 8-bit samples (*“image_conversion.ipynb”*). It incorporates options to apply sharpening and contrast enhancing in terms of intensity rescaling and contrast limited adaptive histogram equalization (CLAHE), enabled by the Python “scikit-image (skimage)” package^41^.

The GRAZPEDWRI-DX dataset enables assessment of a variety of research questions around the injured pediatric wrist. To our knowledge, there are no related pediatric datasets publicly available. In adults, there is a limited number of musculoskeletal radiography collections available to the community. The Stanford University datasets MURA^25^ and LERA^42^ contain large numbers of samples but feature only binary labels, in terms of “normal” and “abnormal”. The MURA dataset consists of 14,863 studies (total of 40,561 multi-view radiographic images) from 12,173 patients^25^. The LERA dataset accumulated lower extremity radiographs of 182 patients^42^. Both lack comprehensive labeling that we provide within the current dataset.

We might release further revisions of the dataset annotations in the future.

## Data Availability

All anonymization steps were computed in Python 3.8.2 on a Windows 8.1 platform as described in the Methods section. We are not able to publicly share the actual code involved in the de-identification procedure, as patient information was processed. The de-identification procedure should be sufficiently reproducible based onto the presented information. We attached a Jupyter notebook that is able to preview images including tags and labeled objects (*“annotation_preview.ipynb”*). It can be found in the “notebooks” folder and online at Figshare under 10.6084/m9.figshare.19330688.v1^43^. The dataset is accompanied by a Jupyter notebook that is able to split files into different folders, based onto two columns of a CSV file (*“copy_files_by_csv.ipynb”*). It is intended to efficiently prepare datasets for image classification tasks with the included “dataset.csv” file. Image post-processing code is publicly provided as second Jupyter notebook with the dataset (*“image_conversion.ipynb”*), also located in the “notebooks” folder.
